# Experiences of hearing loss and views towards interventions to promote uptake of rehabilitation support among UK adults

**DOI:** 10.1080/14992027.2016.1200146

**Published:** 2016-07-05

**Authors:** Crystal Rolfe, Benjamin Gardner

**Affiliations:** ^a^ Department of Epidemiology and Public Health, University College London, UK; ^b^ Action on Hearing Loss, London, UK; ^c^ Department of Psychology, Institute of Psychiatry, Psychology and Neuroscience, King’s College London, London, UK

**Keywords:** Assistive technology, behavioural measures, hearing aid satisfaction, psycho-social/emotional

## Abstract

*Objective*: Effective hearing loss rehabilitation support options are available. Yet, people often experience delays in receiving rehabilitation support. This study aimed to document support-seeking experiences among a sample of UK adults with hearing loss, and views towards potential strategies to increase rehabilitation support uptake. People with hearing loss were interviewed about their experiences of seeking support, and responses to hypothetical intervention strategies, including public awareness campaigns, a training programme for health professionals, and a national hearing screening programme. *Design*: Semi-structured qualitative interview design with thematic analysis. *Study sample*: Twenty-two people with hearing loss, aged 66–88. *Results*: Three themes, representing barriers to receiving rehabilitation support and potential areas for intervention, were identified: making the journey from realization to readiness, combatting social stigma, and accessing appropriate services. Barriers to receiving support mostly focused on appraisal of hearing loss symptoms. Interventions enabling symptom appraisal, such as routine screening, or demonstrating how to raise the topic effectively with a loved one, were welcomed. *Conclusions*: Interventions to facilitate realization of hearing loss should be prioritized. Raising awareness of the symptoms and prevalence of hearing loss may help people to identify hearing problems and reduce stigma, in turn increasing hearing loss acceptance.

## Introduction

In the UK, hearing loss (HL) is thought to affect around 11 million people (Davis, 1995; RNID Action on Hearing Loss, 2015). Auditory deprivation affects understanding of speech (Welsh & Purdy, 2001), which can prompt social withdrawal, isolating people with HL and their partners (RNID Action on Hearing Loss, 2010). Perhaps consequently, HL is associated with poorer health-related quality of life, emotional distress, and depression (Chia et al, 2007; Saito et al, 2010; Gopinath et al, 2012). HL can also indirectly affect health due to difficulties accessing and comprehending medical advice. This is particularly important as half of older people—the demographic in which HL is most prevalent (Davis et al, 1995)—have other disabilities and long-term health conditions (RNID Action on Hearing Loss, 2015).

Hearing aids (HAs) are the most common rehabilitation option for HL, and within the UK, high-quality digital HAs are available free on the National Health Service. Other forms of rehabilitation, such as assistive devices and lip-reading classes, are free from some UK local authorities. HAs and assistive devices can also be purchased privately from high-street retailers. Early uptake of such interventions can improve quality of life (Chisolm et al, 2007). Yet, many do not receive rehabilitation support, or face considerable delay. For example, only a third of people with HL have HAs, and people typically experience an average delay of 10 years before receiving HAs (Davis et al, 2007). Behaviour change interventions are needed to increase early uptake of rehabilitation support.

Developing effective rehabilitation support promotion interventions depends on understanding factors that determine avoidance or delay among people with HL. Theoretical models of delay organize factors determining rehabilitation uptake (Manchaiah et al, 2011). For example, Walter et al, (2012) distinguish delays in: *appraisal*, which focuses on labelling symptoms, and culminates in perceiving a reason to discuss symptoms with a health professional; *help-seeking*, which focuses on deciding to consult a professional and making necessary appointments; *diagnostics*, encompassing the investigations, referrals, and appointments that culminate in diagnosis; and *pre-treatment*, which focuses on planning and scheduling of rehabilitation support. Within each of these intervals, patient, healthcare and system factors, and factors related to HL itself, combine to determine progress towards rehabilitation support (Walter et al, 2012). For HL, delays have been documented at all stages. Many people experience a considerable time lag between initially experiencing HL symptoms and appraising them as warranting professional support (Wänström et al, 2014). Within the help-seeking stage, the perceived stigma of HL, often attributable to perceived associations between HL and ageing (RNID Action on Hearing Loss, 2015), causes delay in almost half of cases (Kochkin, 2007). There may be significant healthcare provider delays in pre-treatment: many practitioners fail to refer to appropriate rehabilitation support services (Davis et al, 2007). Some who have sought help withdraw at the pre-treatment stage (Kochkin, 2007; Meyer & Hickson, 2012). HL rehabilitation uptake could potentially be promoted via intervention within appraisal, help-seeking, diagnostic, or pre-treatment stages (Echalier, 2010; Meyer & Hickson, 2012; Wänström et al, 2014). The effectiveness of such interventions would, however, depend on their acceptability to people experiencing HL; interventions with which target recipients are unwilling or unable to comply are unlikely to prove effective (Craig et al, 2008; Michie et al, 2014). Interventions are likely to be most acceptable where they acknowledge and address the lived experiences of people with HL and potential obstacles to receiving support (e.g. Barker et al, 2016). Potential reasons for delay in receiving rehabilitation support have been well-documented (Meyer & Hickson, 2012), but little work has explored patients’ views towards potential interventions to minimize delay. While 90% of people with HL surveyed by Davis et al, (2007) believed screening for HL to be acceptable, particularly in a GP practice, little is known about the acceptability of alternative rehabilitation options. Using qualitative methods to document views towards interventions among the target population can serve two functions. First, reactions to proposed interventions can inform refinements to those interventions, enhancing feasibility and so likely effectiveness (Craig et al, 2008), or generate ideas for new interventions. Second, analysis of responses to interventions can reveal underlying barriers or facilitators to HL rehabilitation support that may not be revealed through direct questioning.

This study aimed to describe experiences of HL and views around possible intervention strategies to promote uptake of rehabilitation support among UK adults. Semi-structured interviews were used to record patients’ narratives of their HL experiences, and their responses to credible hypothetical intervention strategies.

## Method

### Preliminary work: Generating interventions for discussion

No standardized intervention exists to promote uptake of rehabilitation support within the UK. Two panels were convened to co-design a set of credible hypothetical intervention approaches, targeting various potential sources and stages of delay in support provision, to stimulate discussion in interviews. An ‘expert panel’ comprised ten experts, covering audiology, research, marketing, public relations campaigns, and including a volunteer with HL. The expert panel participated in four workshops facilitated by the first author. They were tasked with identifying discrete target behaviours that would facilitate or inhibit rehabilitation support (e.g. people with HL attending regular hearing checks, GPs referring patients for hearing tests). The COM-B model of behaviour (Michie et al, 2011) and Theoretical Domains Framework (TDF; Cane et al, 2012), which specify core barriers to behaviour change, were used to identify ideas for how to change the identified target behaviours (see also Barker et al, 2016). The feasibility of these ideas was assessed against criteria of affordability, practicability, effectiveness and cost effectiveness, acceptability, safety, and equity (Michie et al, 2014). A ‘lay panel’, comprising three people with HL, convened twice to verify the credibility of expert-generated intervention ideas, facilitated by the first author.

This process generated a set of eight hypothetical intervention strategies, which together targeted various stages of delay, and patient, professional, and system factors. These were: (1) a campaign to encourage and train friends and family in how to sensitively support people with HL to obtain professional help; (2) a campaign to raise public awareness of HL, including stories of people who successfully obtained professional support for HL; (3) a campaign to raise public and professional awareness about the rehabilitation support options available for HL; (4) a national hearing screening programme; (5) rehabilitation support provision occurring concurrently with assessment and diagnosis; (6) improvements to the aesthetic design of HAs and increased availability; (7) increased availability of a greater range of rehabilitation support options, such as devices, lip-reading classes and communication tactics, either as an alternative or complement to HAs; and (8) a national training programme for GPs and other health professionals, to increase awareness of and referral to specialist support available for people with HL.

### Design and participants

A semi-structured interview design was used. Twenty-two participants were recruited from three sources linked to one UK HL charity: three responded to posters and fliers at the headquarters of the charity, ten responded to an email circulated to the charity’s research panel of the charity, and nine were recruited at community hearing support sessions hosted by the charity. Participants were included if they were aged over 55, spoke English fluently, and had been diagnosed with and accessed rehabilitative support for HL. Participants self-verified eligibility before, and completed a brief questionnaire after, the interview. Questionnaire measures included age, gender, ethnicity, education, time taken to access rehabilitation support from when HL first noticed, support obtained, and whether participants had any disability (other than HL) or long-term illness. Approval was granted by the < ANONYMISED > ethics committee (ref 5805/001).

Participant age ranged from 66–88 years (mean 74, SD 1.7; median 73.5 years). Thirteen participants were female. Fifteen were White British, three Indian, two White Irish, one White Other, and one Asian Other. Eleven had higher education or equivalent qualifications, two had O-Level or vocational equivalents, two ‘any other’ qualifications, and five no formal qualifications. Participants self-reported taking between 1–5 years from first noticing to receiving support for their HL (mean 2.5, SD 1.3; median 2 years; Table 1). All participants obtained HAs as their main form of rehabilitative support, 14 receiving HAs only, and eight also having used devices, taken lip-reading classes, and/or received ear surgery. Six had another disability or long-term illness, most commonly diabetes, back pain, heart, or mobility problems, of whom three had multiple conditions.

**Table 1.** Participant characteristics.

Participant number	Non-HL disability or long term illness?	Time from first noticing HL to receiving support (years)	Rehabilitation support obtained
1	No	2	HAs
2	Yes	2	HAs
3	Yes	5	HAs
4	No	2	HAs & lip-reading classes
5	Yes	3	HAs
6	No	2	HAs
7	No	2	HAs & lip-reading classes
8	No	5	HAs & lip-reading classes & ear surgery
9	Yes	2	HAs
10	No	2	HAs
11	No	2	HAs & lip-reading classes & devices
12	No	1	HAs & Lip-reading classes & devices
13	No	2	HAs & lip-reading classes
14	No	2	HAs
15	No	5	HAs & devices
16	Yes	2	HAs
17	No	1	HAs & lip-reading classes
18	Yes	2	HAs
19	No	5	HAs
20	No	4	HAs
21	No	2	HAs
22	No	1	HAs

### Procedure and interview schedule

Interviews were conducted face-to-face, lasting between 30–50 minutes. Participants were encouraged to talk freely, with an interview schedule used to ensure coverage of key topics. Each interview was initiated by discussing personal experiences of HL, and the support participants sought and received. After this, written descriptions of the eight interventions were presented, and participants were asked to comment on their potential acceptability.

### Analysis

For indicative purposes, the acceptability of each of the eight intervention strategies was coded (see Table 2). An intervention was deemed ‘acceptable’ where a participant explicitly stated that it would be helpful or useful.

**Table 2.** Acceptability of hypothetical interventions.

Intervention description	Acceptable	Not acceptable	Not stated
(1) Encouraging and training friends and family in supporting people with HL to obtain rehab support	13	2	7
(2) Raising public awareness of HL	9	8	5
(3) Raising public and professional awareness of rehab support options	9	2	11
(4) National hearing screening programme	15	1	6
(5) Rehab support provided concurrently with assessment and diagnosis	3	4	15
(6) Improved design and availability of hearing aids	9	7	6
(7) Increased availability of range of rehab support options	1	12	9
(8) National training programme for health professionals	11	2	9

Intervention strategies were coded as ‘acceptable’ to a participant where the participant made explicit statements that the strategy would be helpful or useful.

Verbatim transcripts of digital interview recordings were analysed using inductive thematic analysis, with realist epistemological assumptions (Braun & Clarke, 2006). Transcripts were read repeatedly, and initially coded line-by-line to assign conceptual labels to excerpts deemed relevant to our two research aims. Labels were refined using constant comparison (Glaser & Strauss, 1967), and excerpts relabelled where necessary. Conceptual labels were organized into clusters, to form coherent themes (Braun & Clarke, 2006).

The first author coded all data. For verification purposes, two transcripts were independently coded by the second author (an experienced qualitative researcher), and disagreements resolved through discussion. Regular meetings were held between both researchers to verify the coherence of emergent themes, and that examples were illustrative of themes, so ensuring appropriate interpretation of data (Mays & Pope, 1995).

## Results

The most acceptable interventions were a national hearing screening programme (acceptable to 15 of 22 participants), encouraging friends and family to support people with HL (13/22), and a health professional training programme (11/22). Three themes were identified, representing potential barriers to seeking rehabilitation support for HL, and subsuming individual experiences of HL and views towards interventions. These were: making the journey from realization to readiness, combatting the social stigma of HL, and accessing appropriate services.

### Making the journey from realization to readiness

Five participants stated, prior to being shown the interventions, that nothing could have helped them to access rehabilitation support before they were ready, as the decision to seek support ultimately had to be self-generated:

Participant 21 (P21): *[Family] can’t do anything until you say ‘I think I’ve got a problem’.*

There were several commonalities in participants’ recollections of their journey to a state of ‘readiness’. Most initially misattributed symptoms of HL to external factors.

P2: *I just put [my HL] down to where I was working…I thought it [the problem] was [due to] them [i.e. the person talking to me], not me … Just thinking [that the lack of birdsong was because there were] not many birds in the garden today, or something like that.*

For many, diminished hearing capacity was realized through using everyday sounds as objective indicators, or through comparing their hearing to that of others.

P1: *[For me] it was particularly the television, because family members could have it at volume 10 and I could have it about 22. […] [And] when you’re driving a car, the indicators … you can’t hear [them] and then you think ‘yes, there is something amiss here’.*

Some felt that encouraging people to monitor their hearing capacity against objective criteria could raise awareness of declines and hasten self-realization.

P10: *Make [people with HL] ask themselves ‘Am I alright?’ … [For example,] how many times did I say ‘pardon?’ to a person in a matter of half an hour.*

Participants typically modified their environments so as to offset HL symptoms, and only became ready to seek help when they realized their HL could not be mitigated nor would it remedy itself.

P11: *Some days … it seemed to be worse than others […] I was having a bad day and I thought this isn’t going to go away by itself, do something.*

For some, social withdrawal impeded detection of HL *(‘[I might have sought help sooner] if I had joined in with other things and realized earlier that I was missing out on such a lot’;* P22*)*. Conversely, most accepted they needed help when communication suffered:

P15: *You don’t necessarily like to admit it to yourself that you have a problem [… but] my husband had a health problem […] and I* thought*, supposing … he had a collapse or something? I thought if I am having problems hearing this could make life much worse. I have really got to do something.*

Even when patients had reached a state of ‘readiness’, action was often delayed further because HL was not prioritized over competing day-to-day demands:

P22: *I put things off. I’ve got lots of other things that I prefer doing.*

An intervention based on promoting action among family members to encourage people with HL to seek and prioritize support was mostly deemed acceptable. Yet, the style of communication that participants’ own families used to raise concerns often prompted resistance *(‘well in my case it wasn’t encouragement, [my family] were just being plain rude’;* P7*)*.

P14: *The more [my wife] nagged the less I wanted to do anything…eventually through enough nagging I went and got … hearing aids.*

Others thought family were reluctant to raise the topic of HL for fear of causing offence, though ironically, participants were generally more offended at suspected HL deliberately *not* being raised by others. Many felt that discussions initiated sensitively might have encouraged their help-seeking.

P12: *I think they [family] could have been a bit more open about it. They didn’t want to offend me … they didn’t say it to me, they just said it* to *my wife […] if we’d actually had a sit down talk about it [I might have sought support]. No-one did, it was just a passing comment and I was always hostile to those suggestions.*

Others felt that speaking to people with HL could stimulate action:

P15: *If I had known someone that had hearing aids, somebody had spoken to me who actually had it themselves, I am sure that kind of personal, one to one thing [would have encouraged me to seek help].*

For many, offers of support from qualified healthcare professionals were important in making the journey to self-realization *(‘the practice nurse suggested a test…, I just said “yes, go for it”’*; P2), with GPs particularly trusted due to their expertise:

P4: *He [GP] said that the sooner you [get HAs], the sooner you will get used to it. I accepted his advice rather than quibbling. Well he’s an expert … so I accept his knowledge.*

Most participants said they would have attended a screening invitation *(‘[It would be] like breast screening. You go because you are supposed to, whether you want to or not’;* P13). Screening was acceptable to most, and most acceptable where embedded in other health checks, which was expected to maximize the chance of signposting to appropriate services:

P16: *I think it would be better, when the doctor starts to check for blood pressure, to do a quick check at least of your hearing, and then they could send you to the hospital if they thought it was necessary.*

Self-screening, using tests available online or by phone, was less popular as participants felt that professional involvement was important to validate and encourage acceptance of test results, and prompt action.

P2: *You [would just] test yourself, say ‘oh well, that’s it, I’m going a little bit deaf’, [and] just walk away.*

### Combatting the social stigma of hearing loss

Even after accepting HL, participants were reluctant to identify as a person with HL, associating HL with ageing, disability, and prosthetics. Many sought to hide HL from others, for fear of disapproval or otherwise differential treatment.

P13: *I didn’t want my employer to think that I was wearing hearing aids and getting old.*

The visibility of HAs delayed participants getting help, and many expressed a strong preference for hidden HAs *(‘I thought if they are going to offer me big things [hearing aids], that [option is ruled] out’; P20).* Some people felt pressure from others to conceal HAs (‘*Everybody that knows I have got one of these say “aren’t they big! Can’t you have a smaller one?”’; P18)*. Perhaps consequently, interventions based on improving HA design were identified by half of participants as most likely to have encouraged them to seek support for HL. Ironically, HA marketing aimed at increasing uptake by emphasizing that HAs can be hidden was viewed by some as perpetuating stigma, and some felt that enhancing HA visibility could destigmatize HAs and HL.

P21: *They had this full page ad about how you can’t even see it, as though it’s something that’s always got to be hidden away […] You don’t tell people who wear glasses to [hide them] … you don’t tell people who can’t walk to hide their legs.*

P11: *The whole emphasis seems to be try to make them tiny, un-noticeable, and you can’t. They are noticeable, so why not make them bright colours, patterns. … It is making it not a fuddy duddy, elderly thing.*

Others preferred hidden HAs however, despite recognizing that they could remove visual cues to HL for other people:

P12: *I would prefer if they were [invisible] but at the same time they are a warning to people around you that [you] can’t hear.*

Some people delayed seeking help because they did not want to feel part of a minority group, and so felt that raising awareness of the prevalence of HL and HAs would be beneficial:

P1: *HAs are not widely worn by the public… The information should be that there are a lot of people in this country who are hard of hearing and they have benefited by getting a HA.*

Some reported that making comparisons with celebrities with HAs was encouraging and boosted self-esteem by reducing stigma:

P17: *There is nearly always a celebrity featured [in the hearing charity magazine]. It’s often a surprise [to me] as to who has got a HL and it did encourage me [to seek help].*

Some felt that improving the visual or social image of HAs would encourage help-seeking.

P20: *if I had known that HAs weren’t quite so ugly looking as what we all thought they were…., that would have definitely helped.*

One participant spoke of a lack of visible, factual coverage of HL in the media (‘*you hardly see any documentaries about hearing loss;’* P1), and another drew a parallel to the positive impact of celebrity endorsement on the social acceptability of glasses:

P11: *When I was at school, glasses were horrible. They were not things cool kids wore until John Lennon and his little round glasses, and suddenly they were alright.*

Others felt that increasing the visibility of HL among everyday public figures, with whom they could more easily identify, would have had a beneficial impact on their own support-seeking.

P13: *Celebrities don’t impinge on me hugely, but … local people in the community, politicians, journalists [do].*

### Accessing appropriate rehabilitation support

Participants reported that HL was rarely proactively raised by GPs (‘*unfortunately GPs … only tackle what you tell them to tackle’;* P1), but onward referral to secondary care had been prompt when requested.

P16: *When I went to ask him [GP] about it, he checked it and … straight away referred me to the hospital … but up until then – well they don’t go round looking for problems.*

Treatment costs caused many participants to delay help-seeking, as they were concerned that they would have to forfeit pleasurable activities in order to buy them privately; many had not realized that NHS HAs were free *(‘I never thought of going National Health; I didn’t realize these were free’*; P12). Emphasizing the low-cost of support might therefore increase uptake:

P14: *Privately, the reason one won’t go is because you already think ‘This is going to be bloody expensive; they’re going to sell me something’. Making it obvious that [there’s] … no commitment [to buy HAs would help].*

Many felt it would be more appealing to seek support if services were more visible and so easier to access.

P1: *There is a lack of awareness as to where people can go and get help because … you know it’s not a high street setup… most audiologists are hidden somewhere … if it’s next door to a GP’s practice it’s much easier.*

Despite general dissatisfaction with waiting times, participants reported that they had typically committed to obtaining support once the process was underway, due to sunk costs incurred by first seeking professional help.

P20: *I thought ‘Leave it, don’t bother’. But then I thought … ‘I have gone this far. I might as well see it through’… I don’t think that matters [assessment and fitting together]. You don’t expect to get something done bang right away.*

Alternative rehabilitation options to HAs were generally unappealing, as they were seen as less effective and time-consuming *(‘I did lip reading for 2–3 years and I don’t think I got anything at all from it’;* P7), as well as more stigmatized *(‘I associate things like lip reading … with profoundly deaf people’;* P22). Their promotion as a substitute for HAs was generally not an acceptable rehabilitation support promotion intervention, though some reported that they would be more willing to use alternative devices if they were normalized and available from everyday locations.

P11: *You may not stand and read [a sign for HL rehabilitation products], but it is in there [products for HL in high street shops], you see you can get these things, and it would also help in improving the image, because it wouldn’t be separate from things for normal people.*

For some, knowledge of the benefits of HAs had been an important factor in deciding whether to seek support. Allowing people to experiment with HAs was viewed as an acceptable means to increase motivation to get them, so reducing delay in seeking support.

P20: *If they had HAs […] you can try and see what difference it makes to you. You can’t go around saying to somebody ‘Can I have your hearing aids?’ to try and see if it makes any difference to me.*

## Discussion

The views expressed by our sample generate ideas for promoting rehabilitation uptake. Many did not initially recognize their HL, and so interventions facilitating appraisal of HL symptoms were viewed positively. While many felt stigmatized by HL, views towards interventions to address stigma were mixed. Some believed that reducing hearing aid (HA) visibility would avoid stigmatization, while others felt that interventions should instead seek to destigmatize HAs and HL. These findings highlight barriers towards implementation interventions and possible means of overcoming them.

Models of patient delay describe multiple stages between initial symptom appraisal and receiving appropriate support (Walter et al, 2012). While some concerns were raised about the visibility and accessibility of professional HL support services, participants predominantly reported that their receiving support was delayed by failure to accurately appraise symptoms, rather than healthcare system factors (see also, Rawool & Keihl, 2008). Participants did not recognize HL because they were able to mitigate symptoms or misattribute them to external factors. Interventions aimed at increasing realization were acceptable. A national screening programme that would objectively verify HL, and direct them towards specialist support was welcomed. Such a programme could potentially increase rehabilitation support uptake among those with HL (Chou et al, 2011), while removing the onus on the person experiencing symptoms to proactively seek help. Screening is endorsed by the US Preventive Services Task Force (Yueh et al, 2003), but a recent consultation concluded there was insufficient high-quality evidence to justify introducing such a programme in the UK (UK National Screening Committee, 2016). Nonetheless, our results support previous findings in demonstrating that screening would be publicly acceptable (Davis et al, 2007). Effective self-screening methods are available as a cheaper alternative to population-based screening (Smits & Houtgast, 2005), but our participants found self-screening less acceptable because of concerns about insufficient post-test support. Screening administered by health professionals, as part of a suite of health checks, was most acceptable. Self-administered, objective hearing tests might be viewed more positively if complemented by appropriate and timely support, such as automated transfer of results to health professionals.

Some participants felt family members could have offered more help in obtaining timely support. Yet, those encouraged by family to seek support often found discussions about HL demotivating (see also, Echalier, 2010). This apparent contradiction may be resolved by recognizing the importance of intrinsic motivation in stimulating action (Ryan & Deci, 2000). People who are intrinsically motivated (i.e. driven by self-generated motives) tend to be more strongly motivated per se, and are more likely to act on their motivation, than those extrinsically motivated (driven by external pressures; Hagger & Chatzisarantis, 2008). Indeed, several participants were unwilling to seek support until they felt personally ready—i.e. intrinsically motivated—to do so. By contrast, as our results testify, external pressures can prompt psychological reactance, whereby people show strong adverse emotional reactions to perceived threats to their freedom, and react in a way that reinstates their autonomy (Brehm & Brehm, 1981). The good-willed actions of family members may thus, ironically, reduce the chances of professional support being sought. It is, however, possible for external demands to be internalized. Persuasive communication that supports the competence and autonomy of the recipient is more positively received (Ryan & Deci, 2000). Campaigns should help the public to employ communication strategies that sensitively support, rather than exert pressure, on loved ones with HL.

Many participants associated hearing aids (HAs), and HL more broadly, with ageing and disability, and were reluctant to identify as having HL, or wear HAs. Several participants reported that learning HL was common, or seeing images of others with HL, made them feel more positive about HL and more willing to wear HAs. This echoes previous evidence showing stigma to affect acceptance of HL (Wallhagen, 2009). Past research has shown that the decisions made by people with HL arise from weighting the everyday challenges posed by HL against its threat to self-identity (Wänström et al, 2014). This has important implications for rehabilitation support. Identity generates identity-relevant actions (West & Brown, 2013): reluctance to identify as having HL may make people unwilling to engage with HL rehabilitation support (Kochkin, 2007). Ironically, while participants felt that they should not have to hide their HAs for fear of stigmatization, many nonetheless concealed their HAs for this very reason. This cycle may perhaps be broken by longer-term intervention strategies to make HL more socially acceptable (Barker et al, 2016). Stigma might be combatted by increasing the visibility and public acceptance of HAs and HL among people with whom those with HL can identify. Drawing attention to the prevalence of HL may also help to combat the misperception that HL is uncommon.

Limitations must be acknowledged. Firstly, we sampled people with HL, but the views of other stakeholders, such as audiologists, will also determine the feasibility of HL rehabilitation support interventions (Barker et al, 2016). We attempted to mitigate this by involving a range of stakeholders in generating intervention ideas to stimulate discussion. Secondly, we sampled predominantly White British and well-educated participants. People with higher qualifications are more likely to seek help for HL, and sooner (Laplante-Levesque et al, 2012). Indeed, all participants reportedly accessed support in fewer than the 10 years reported in a larger, more demographically diverse sample (Davis et al, 2007). The accuracy of self-reported delay cannot however be verified; participants may have underestimated delay by basing their estimates on delay from realization of HL, rather than from first observation of symptoms indicative of HL. Thirdly, we focused on those who had sought help. While ideally we would have recruited people experiencing early signs of HL in need of support, it is of course difficult to recruit those who have not identified themselves as experiencing HL. Our data may not represent the views of those less willing to seek help. Lastly, while our results give an indication of some of the likely responses to intervention strategies, it is unclear whether, given our small sample size, we captured all such responses. Relatedly, we assumed that participants accurately recalled their experiences and factors influencing their decisions to seek help, but perceived influences on actions may not reflect true influences (Nisbett & Wilson, 1977). This problem is compounded by our reliance on historical recall; people may falsely recall past action and its determinants (Koriat, 1993).

Nonetheless, this study is the first to our knowledge to document views towards potential strategies to encourage people to seek professional support for HL, as rooted in participants’ own support-seeking experiences. Barriers were predominantly located in appraisal of HL symptoms rather than access to services, and so interventions that seek to enable realization of HL, such as screening and encouragement of appropriate assistance from family, may have most potential for publicly acceptable implementation.
